# Meeting global standards for hand sanitizer efficacy: formulation matters

**DOI:** 10.1186/1753-6561-5-S6-P26

**Published:** 2011-06-29

**Authors:** SL Edmonds, DR Macinga, P Mays-Suko, C Duley, JW Arbogast

**Affiliations:** 1 GOJO Industries, Inc, Akron, USA; 2 BioScience Labs, Bozeman, USA

## Introduction / objectives

Critical questions have been raised in the scientific literature and by hand-hygiene thought leaders regarding the minimum alcohol concentration that assures efficacy of alcohol-based hand rubs (ABHR). The objective of this study was to determine the relative influences of alcohol concentration and product formulation on the efficacy of ABHR using internationally recognized methods.

## Methods

Eleven commercially available alcohol-based hand rubs (gels and foams) containing between 60-90% (v/v) ethanol and WHO-recommended hand rub formulations containing 75% isopropanol or 80% ethanol were evaluated in a series of studies. Test methods included EN 1500 (Hygienic Hand Rub) and ASTM E1174 (Healthcare Personnel Handwash).

## Results

Four ABHR ranging from 70-80% ethanol met EN 1500 requirements with a 3 ml application volume applied for 30 seconds. Nine ABHR and the 2 WHO formulations were evaluated per E1174 at 2-ml application volumes. Of the products tested, only 2 products, a well-formulated 70% ethanol ABHR gel and well-formulated 70% ethanol foam, met the U.S. FDA requirements (reductions of ≥2 log_10_ after 1 application and ≥3 log_10_ after 10 applications). None of the other nine products achieved a 3-log_10_ reduction following the tenth application.

## Conclusion

Product formulation was found to have a greater influence on efficacy than alcohol concentration. Well-formulated products containing 70% ethanol, including ABHR foams, can exhibit greater efficacy than products with higher alcohol levels. These results demonstrate that alcohol concentrations in excess of 70% are neither necessary, nor always sufficient to meeting global efficacy standards.

## Disclosure of interest

S. Edmonds Employee of GOJO Industries, D. Macinga Employee of GOJO Industries, P. Mays-Suko: None declared, C. Duley: None declared, J. Arbogast Employee of GOJO Industries.

